# Consultation Liaison (CL) Psychiatry and Division of Medicine: Collaborating to Pilot a Behaviours of Concern Rapid Response Team (BoC RRT)

**DOI:** 10.1192/j.eurpsy.2023.861

**Published:** 2023-07-19

**Authors:** R. Smyth, T. Wright, C. Daniel, K. Vincent, M. Konrad, B. Huang, A. Smith, K. Gregorevic, B. Cleveland, R. Feiler

**Affiliations:** 1 Consultation Liaison Psychiatry, The Royal Melbourne Hospital; 2 Department of Nursing, School of Health Sciences, The University of Melbourne; 3 Division of Medicine; 4 Quality Improvement, The Royal Melbourne Hospital, Melbourne, Australia

## Abstract

**Introduction:**

Acute clinical deterioration in hospital inpatients can be caused by a range of factors including dementia, delirium, substance withdrawal and psychiatric disturbance, creating challenges in diagnosis, often requiring a management plan with input from multiple disciplines. Staff forums and broader literature have confirmed that healthcare staff working in non-mental health settings, may not be as skilled in recognising and managing early signs of emerging and/or escalating clinical agitation. The BoC RRT is a consultation service within the Division of Medicine and CL Psychiatry. Staffed by Medical Registrars and Mental Health Nurses, the collaboration provides a unique healthcare response to acute general wards. The BoC RRT has been implemented to address the rising number of incidences whereby staff and patient safety are compromised. Using evidence-based skills the team aimed to: respond to episodes of clinical agitation that require an internal security response, assist ward referrals by exploring biopsychosocial contributants to behaviour, develop individual patient support plans and review and reduce restrictive intervention practices.

**Objectives:**

To determine if the rapid response model has influenced:

- The impact on staff/patient safety

- Frequency of emergency responses for aggression

- Frequency of restrictive intervention use

**Methods:**

This project was approved as a quality assurance project (QA2022018). The patients within scope of the BoC RRT include inpatients in medical and surgical wards. It excludes patients in Emergency Departments, mental health units, outpatient clinics, and visitors. The evaluation of the pilot has used a PDSA (Plan, Do, Study, Act) cycle when implementing new improvements. A mixed methods approach explored the impact of the BoC RRT. Staff consultation will identify challenges in responding to scenarios whereby there is risk of harm to staff and patients. Staff feedback and the emergency response data was monitored.

**Results:**

In 2021, there was approx. 720 code greys per month, requiring a security response. Since the implementation of BoC RRT, these numbers have reduced to 527. Reviewing restrictive intrvention practices has identified areas for policy review and need for education. Staff consultation found that nurses were confident caring for those patients exhibiting clinical agitation associated with delirium and dementia. However, caring for people with mental health or substance use disorders were more challenging.

**Conclusions:**

These interim results indicate that BoC RRT has been generally well received by clinical staff. The decline in code grey responses indicates that it is likely having a positive impact in early identification and management of clinical agitation for hospital inpatients. There is support for this response model to continue beyond the pilot phase and further area for research.

**Disclosure of Interest:**

None Declared

